# Impact of Vesicular Stomatitis Virus M Proteins on Different Cellular Functions

**DOI:** 10.1371/journal.pone.0131137

**Published:** 2015-06-19

**Authors:** Natalia Redondo, Vanesa Madan, Enrique Alvarez, Luis Carrasco

**Affiliations:** 1 Centro de Biología Molecular Severo Ochoa (CSIC-UAM), Nicolás Cabrera 1, Campus de Cantoblanco, Madrid, Spain; 2 Department of Infectious Diseases, Molecular Virology, University of Heidelberg, Heidelberg, Germany; University of British Columbia, CANADA

## Abstract

Three different matrix (M) proteins termed M1, M2 and M3 have been described in cells infected with vesicular stomatitis virus (VSV). Individual expression of VSV M proteins induces an evident cytopathic effect including cell rounding and detachment, in addition to a partial inhibition of cellular protein synthesis, likely mediated by an indirect mechanism. Analogous to viroporins, M1 promotes the budding of new virus particles; however, this process does not produce an increase in plasma membrane permeability. In contrast to M1, M2 and M3 neither interact with the cellular membrane nor promote the budding of double membrane vesicles at the cell surface. Nonetheless, all three species of M protein interfere with the transport of cellular mRNAs from the nucleus to the cytoplasm and also modulate the redistribution of the splicing factor. The present findings indicate that all three VSV M proteins share some activities that interfere with host cell functions.

## Introduction

Vesicular stomatitis virus (VSV) is the prototype member of the *Vesiculovirus* genus that belongs to the Rhabdoviridae family. VSV contains a single-stranded RNA genome of negative polarity that encodes five proteins: nucleocapsid (N), phosphoprotein (P), matrix (M) protein, glycoprotein (G) and large (L) viral polymerase [1]. The first event during VSV gene expression is the transcription of each viral gene by the RNA-dependent-RNA polymerase, which consists of a complex of L and P proteins bound to the 3′ end of the viral RNA. VSV mRNAs, which are capped at the 5′ end and polyadenylated at the 3′ end [2], are subsequently translated by the host cell machinery to produce all viral proteins that are necessary for the replication of the viral genome and its assembly, and eventual release of new virions. Apart from structural and regulatory roles, these proteins also contribute to the cytopathogenesis associated with VSV infection [3].

The interaction of M protein with the viral ribonucleoprotein complex is essential for packaging of viral RNA and assembly of virions. In addition, M protein is associated with the inner leaflet of the plasma membrane and is involved in the budding of the “bullet-shaped” viral particles [4]. The presence of two late (L) budding domains, PPPY and PSAP, within the first 40 amino acids of the N-terminal region of the M protein, contributes to virus egress from infected cells. Recent studies have shown that the PPPY and PSAP motifs mediate the recruitment of host cell factors, E3 ubiquitin ligase Nedd4 and Tsg101, respectively, which are components of the ESCRT1 (endosomal sorting complex required for transport 1) complex, and are required for the late step of virus budding (i.e. the fission between the viral and cell membrane) [5–7].

M protein plays multiple roles in VSV infection, and is the viral component responsible for the majority of the cytopathic effects observed in infected cells. A previous study by Jayakar et al. reported that the M gene encodes two additional polypeptides, denoted M2 and M3, in addition to the 229-amino acid long full length M protein (referred to as M1) [8]. M1 and the smaller M2 and M3 proteins are generated from the same ORF by a mechanism of translation initiation that involves alternative utilization of downstream AUG codons that encode methionine at positions 33 and 51. These shorter forms of M1 protein share an identical C-terminal amino acid sequence and induce cell rounding, a cytophatic effect that leads eventually to death of VSV-infected cells [8]. Apart from their involvement in viral cytopathogenesis, the function of M2 and M3 remains largely unknown. Other cytopathic effects triggered by M1 during VSV infection include disorganization of the cytoskeleton, inhibition of cellular gene expression and induction of apoptosis [9–14]. The blockade of host gene expression by M1 protein has been shown to occur at multiple levels, e.g. M1 inhibits transcription and nuclear export of different RNAs [15–17]. Translation of host cell proteins is also affected during VSV infection [18]; however, the fact that this is not observed when M1 is expressed in the absence of the other viral proteins suggests that inhibition of protein synthesis is a consequence of the suppression of both transcription and mRNA transport, rather than a direct effect of M1 [10, 19, 20]. Although a number of studies have described multiple roles for M1, there is still no evidence for a functional contribution of M2 and M3 proteins. In the present study, we carried out a comparative analysis designed to assess the involvement of M2 and M3 viral products in the functions ascribed to full length M1 protein. We found that alternative expression of shorter forms of M1 is likely not involved in the final step of virus budding, but rather induces cell rounding and partially inhibits translation in cells susceptible to VSV infection. These cytopathic effects mediated by all the three M proteins correlate with a block of cellular mRNA export from the nucleus to the cytoplasm and a selective alteration in the nuclear localization of hnRNP H, a host factor involved in mRNA splicing.

## Results

### Expression of VSV M1 protein and two additional translation products, M2 and M3, in a cell free-system and in BHK-T7 cells

**Fig 1:**
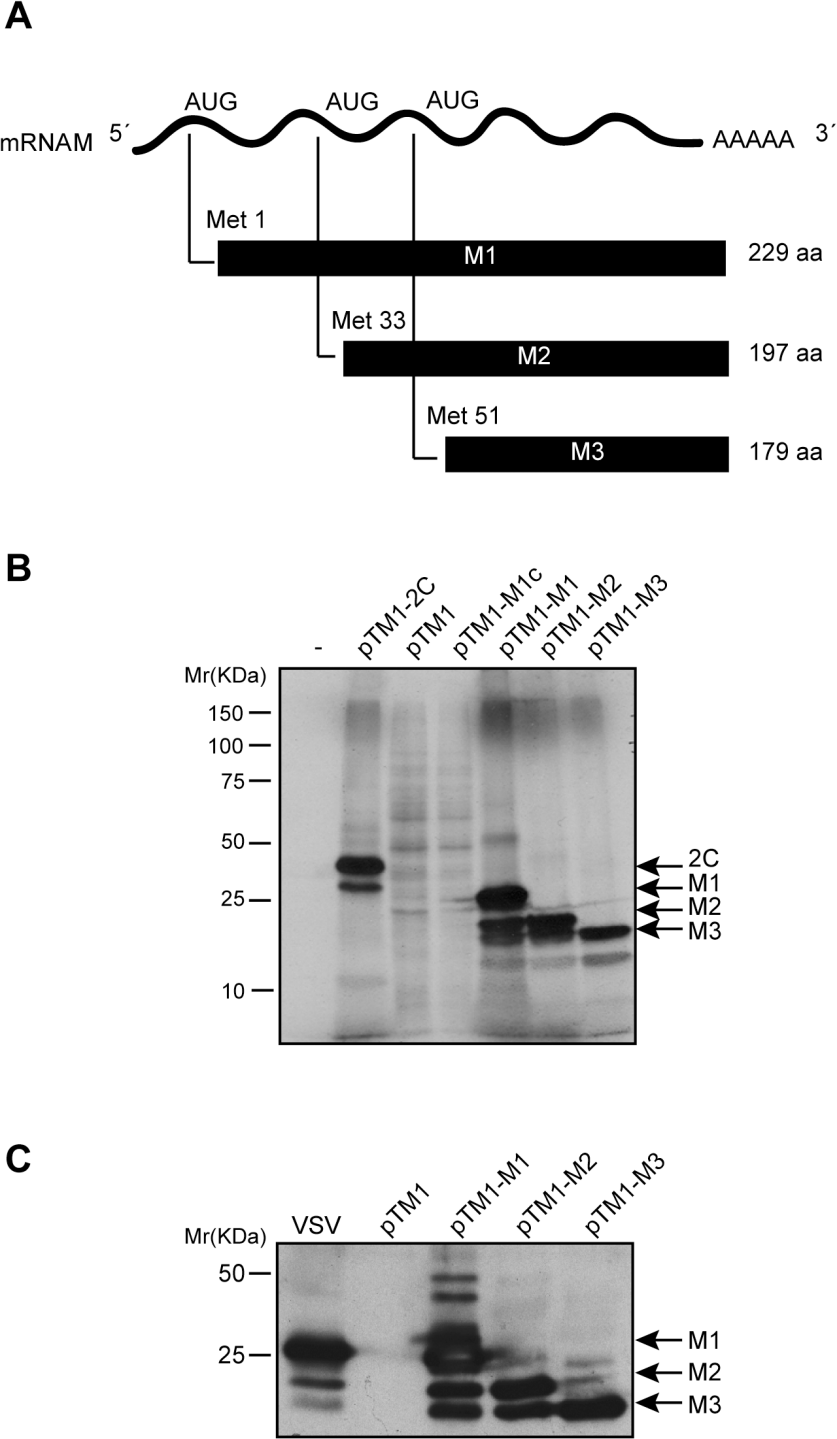
Expression of VSV M1, M2 and M3 proteins in a cell-free system and in mammalian cells. (A) Schematic representation of M mRNA and M1, M2 and M3 proteins of VSV. (B) Translation of VSV M proteins in rabbit reticulocyte lysates. 100 ng of mRNA obtained by *in vitro* transcription from constructs pTM1-M1, pTM1-M2 and pTM1-M3 was incubated with RRLs at 30°C for 2 h with [^35^S]Met/Cys. mRNAs obtained from pTM1-2C (encoding poliovirus 2C protein) or a pTM1-empty plasmid were used as positive and negative controls, respectively. A plasmid no linearized (pTM1-M1c) was used as a reaction control. Protein expression was analyzed by SDS-PAGE (15%), fluorography and autoradiography. (C) Expression of VSV M proteins in BHK-T7 cells. Cells were transfected with pTM1-M1, pTM1-M2 or pTM1-M3 for 2 h, or were infected with VSV at a multiplicity of infection of 10 pfu/cell in DMEM without serum for 1 h at 37°C. Subsequently, the medium was removed and both infected and transfected cells were incubated with DMEM containing 5% FCS at 37°C. Cells were lysed 6 h later and expression of viral proteins was analyzed by Western blot using a mouse monoclonal antibody specific for M1 protein that also recognizes M2 and M3 proteins. Specific products with molecular weight higher than that of M1 might represent oligomeric forms of M proteins. Bands corresponding to different M proteins or poliovirus 2C are indicated with arrows.

It was previously demonstrated that M2 and M3 proteins are not cleavage products from M1 protein, but rather they are synthesized from the M mRNA by alternative initiation at internal AUG codons [8]. To investigate the function of VSV M2 and M3 polypeptides in mammalian cells, we utilized different expression systems. The first goal of our study was to analyze the expression of the three VSV M proteins from M mRNA in a cell-free system. Three plasmids were constructed using the pTM1 backbone vector that contains the T7 polymerase promoter and the encephalomyocarditis virus (EMCV) internal ribosome entry site (IRES), followed by sequences encoding either the full-length M1 protein, M2 (commencing at the AUG encoding Met33) or M3 (commencing at the AUG encoding Met51) (Fig 1A). These plasmids were designated pTM1-M1, pTM1-M2 and pTM1-M3. The corresponding mRNAs encoding the different M proteins were obtained by *in vitro* transcription and subsequently used for *in vitro* translation assays in RRL. The plasmid pTM1-2C, encoding the poliovirus (PV) 2C protein, and the empty plasmid, pTM1, were used as positive and negative expression controls, respectively. Results showed that M1 mRNA preferentially directed the synthesis of M1; however, M2 and M3 protein synthesis was also evident, albeit at lower levels (Fig 1B). Moreover, translation of M2 mRNA largely directed the synthesis of a protein with an identical migration pattern as the M2 protein obtained from M1 mRNA, and also the synthesis of a smaller product with the size expected for M3. Further, as observed for M2 synthesis, the expression of M3 from M3 mRNA was more robust as compared with that obtained from M2 mRNA (Fig 1B). These results are consistent with expression of M2 and M3 by alternative initiation at AUG codons encoding Met-33 and Met-51. Subsequently, the expression of the three M proteins was tested in mammalian cells. BHK-T7 cells, which are susceptible to VSV infection and constitutively express the T7 RNA polymerase, were employed. BHK-T7 cells were transfected with the plasmids encoding M1, M2 and M3, or empty pTM1 as a control, and the synthesis of M proteins in BHK-T7 and in VSV-infected cells was compared. Expression of the VSV M proteins was analyzed by Western blot using a mouse monoclonal antibody specific for VSV M1 protein that also recognizes M2 and M3 [21]. As illustrated in Fig 1C, M1 and the alternative products, M2 and M3, were expressed efficiently from the corresponding transfected plasmids in BHK-T7 cells and the quantity of M1 present was similar to that observed in VSV infected cells at 7 hpi. However, the quantity of M2 and M3 protein synthesized in VSV infected cells was much less than that obtained when pTM1-M2 or pTM1-M3 were transfected (Fig 1C).

**Fig 2:**
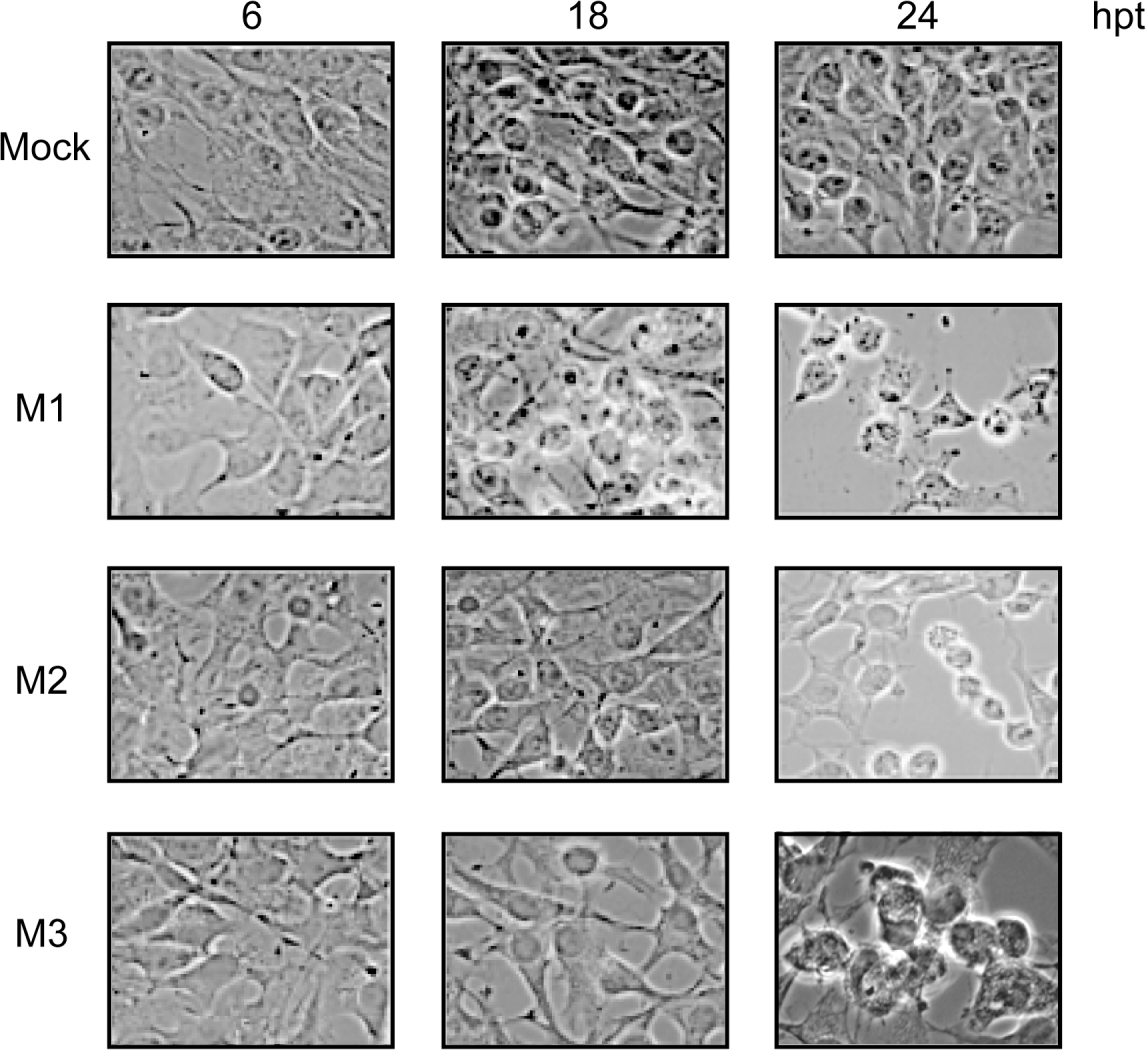
Cytotoxic effect mediated by expression of VSV M proteins. BHK-T7 cells were transfected with pTM1 empty (mock), pTM1-M1, pTM1-M2 or pTM1-M3 for 2 h. Cells were then washed and incubated in DMEM containing 5% FCS until they were fixed at 6, 18 and 24 h post transfection (hpt). Cell morphology was examined with a phase-contrast microscope.

Cytotoxicity associated with VSV infection has been ascribed to the expression of M1 and also to M2 and M3 [8]. To assess the degree of cytotoxicity induced by the synthesis of M1, M2 and M3 proteins in BHK-T7 cells, we studied the morphology of transfected cells at different time points by optical microscopy. Consistent with previous results, the expression of each M protein correlated with the induction of cell rounding and detachment of transfected cells (Fig 2).

### Membrane permeabilization by VSV M1, M2 and M3

**Fig 3:**
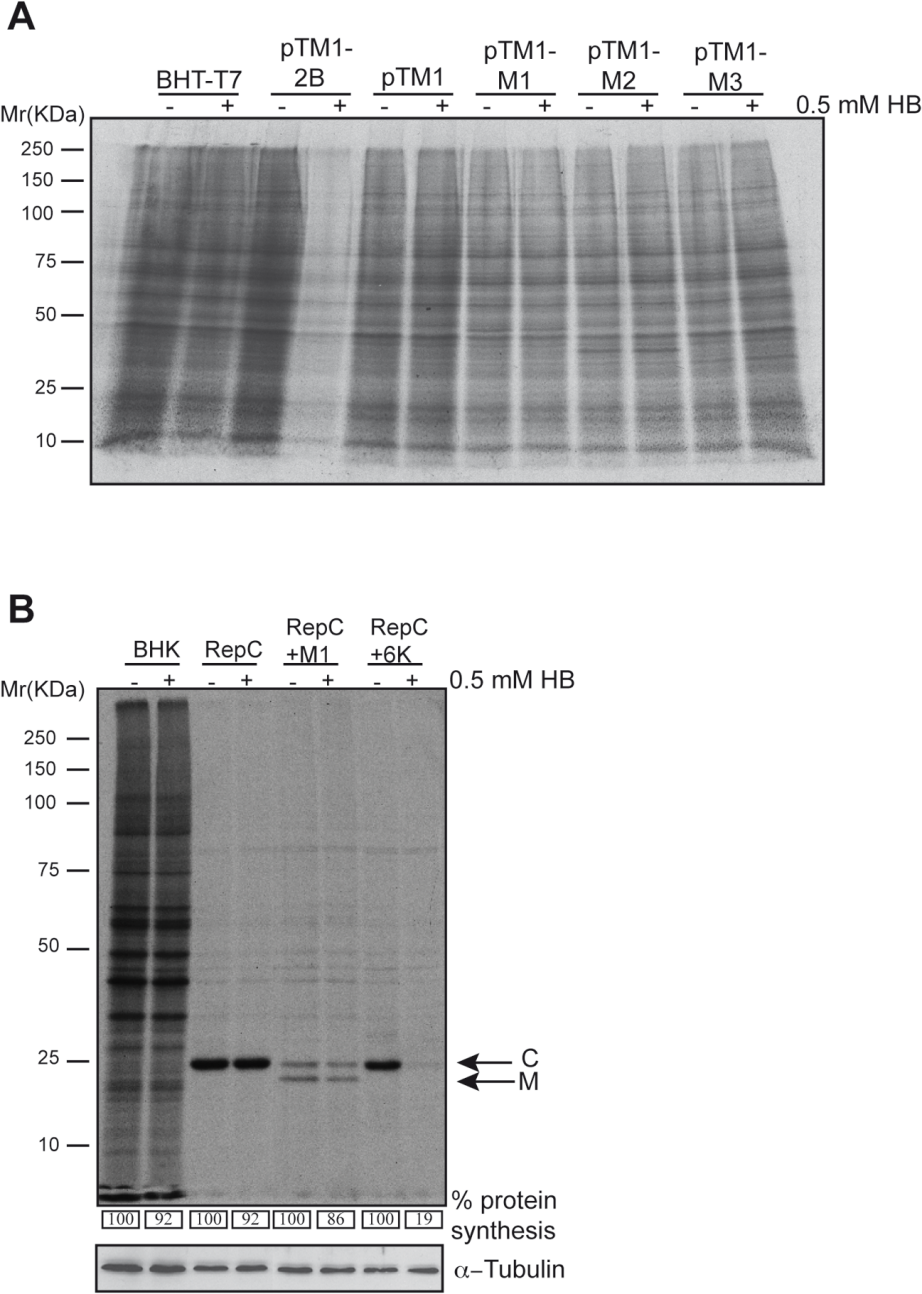
VSV M proteins do not alter cell membrane permeability. (A) BHK-T7 cells were transfected with pTM1 empty plasmid or pTM1 constructs encoding M1, M2 or M3 proteins. As a positive control, cells were transfected with pTM1-2B (encoding poliovirus 2B viroporin). The medium was removed 2 h later and fresh DMEM containing 5% FCS was added. Cells were pre-treated 15 h after transfection with 0.5 mM of the translation inhibitor hygromycin B (HB) for 15 min. Then, cells were metabolically labelled with [^35^S]Met/Cys for 45 minutes in the presence or absence of HB. Samples were processed by SDS-PAGE (17.5%), fluorography and autoradiography. (B) BHK-21 cells were mock transfected or electroporated with SV-derived mRNA replicons: Rep C, Rep C+M1 or Rep C+6K, obtained by *in vitro* transcription from their corresponding DNA templates. Cells were pre-treated with HB and metabolically labeled as indicated in (A). Numbers below each lane indicate the percentages of protein synthesis calculated by dividing the densitometric values for HB-treated cells by the values for untreated cells. A cellular protein band in mock transfected cells, or the band corresponding to the SV C protein in replicon transfected cells, was quantified by densitometric scanning, respectively. Bands corresponding to SV C and VSV M1 protein are indicated with arrows. Detection of α-tubulin served as loading control.

Cell rounding is typically provoked by certain viral proteins that are highly cytotoxic and is particularly evident with membrane-active proteins. We therefore examined the impact of M protein expression on membrane functions such as the maintenance of membrane permeability and the induction of vesicle budding at the plasma membrane. VSV M protein is involved in virus budding, as occurs with a family of viral proteins known as viroporins [22, 23]. Thus, viroporins possess ion channel activity upon their oligomerization, and assemble to form pores in cell host membranes. This activity alters membrane permeability and it is important for virus budding [22, 23]. VSV M1 interacts with cellular membranes both through a carboxy-terminal and a basic amino-terminal domain, which allows the establishment of electrostatic interactions [24, 25]. Moreover M1 can self-interact, forming oligomers, and is involved in the budding of viral particles at the plasma membrane [25–27]. Therefore, both VSV M protein and viroporins exhibit several analogies, including oligomerization membrane interaction and promotion of virus budding. These parallels with viroporins prompted us to study whether M1, or the alternative M2 or M3 proteins, forms pores in the cell membrane and behaves as a viroporin. To test this, we used the aminoglycoside antibiotic hygromicin B (HB) to determine alterations in membrane permeability. HB does not permeate into cells under normal conditions, but readily crosses the plasma membrane upon viroporin expression, and inhibits protein translation [28]. The HB assay was performed in BHK-T7 cells transfected with plasmids encoding M proteins or PV 2B protein, an acknowledged viroporin that was used as a positive control. As expected, the PV 2B viroporin induced potent membrane permeabilization to HB, detected as a drastic reduction in protein translation (Fig 3A). However, this effect was not observed upon expression of M1, M2 or M3, or in control cells transfected with pTM1 (Fig 3A). To further assess membrane permeabilization, an additional approach based on Sindbis virus (SINV) replicons was used [29]. In this system, the sequence of the protein of interest (X) is cloned in-frame downstream of the SINV capsid protein (C), which possesses autoproteolytic activity. The shut-off of cellular translation by the non-structural proteins of the SV replicon is followed by the synthesis of a subgenomic RNA that encodes the precursor C-X. Thus, upon expression and cleavage of C-X, both the SV C and the protein immediately downstream are present in equimolar amounts. This approach allows for a simple quantification of SINV C protein as a metric to determine the entry of HB exclusively in cells that express the protein of interest. To test membrane permeabilization by VSV M proteins, the corresponding SINV replicon bearing the VSV M1 sequence (Rep C+M1) was constructed. Additionally, SINV replicons encoding SINV 6K viroporin (Rep C+6K) or C alone (Rep C) were used as positive and negative controls of membrane permeabilization, respectively. As shown in Fig 3B, expression of SINV C protein did not affect membrane permeability to HB, whereas SINV 6K viroporin provoked the entry of HB and a resultant strong blockade in translation. Notably, VSV M1 protein did not provoke any significant membrane permeabilization to HB at 16 hpt. These results are in agreement with previous experiments performed using an *in vitro* system with artificial membranes [30] and demonstrate that, in contrast to viroporins, none of the VSV M proteins exhibit an ability to disrupt membrane permeability despite the fact that they are integral membrane proteins. These findings indicate that neither the integration of a viral protein into the plasma membrane, nor its oligomerization capacity, is sufficient to perturb membrane permeability.

### Induction of vesicle budding at the plasma membrane by VSV M proteins

**Fig 4:**
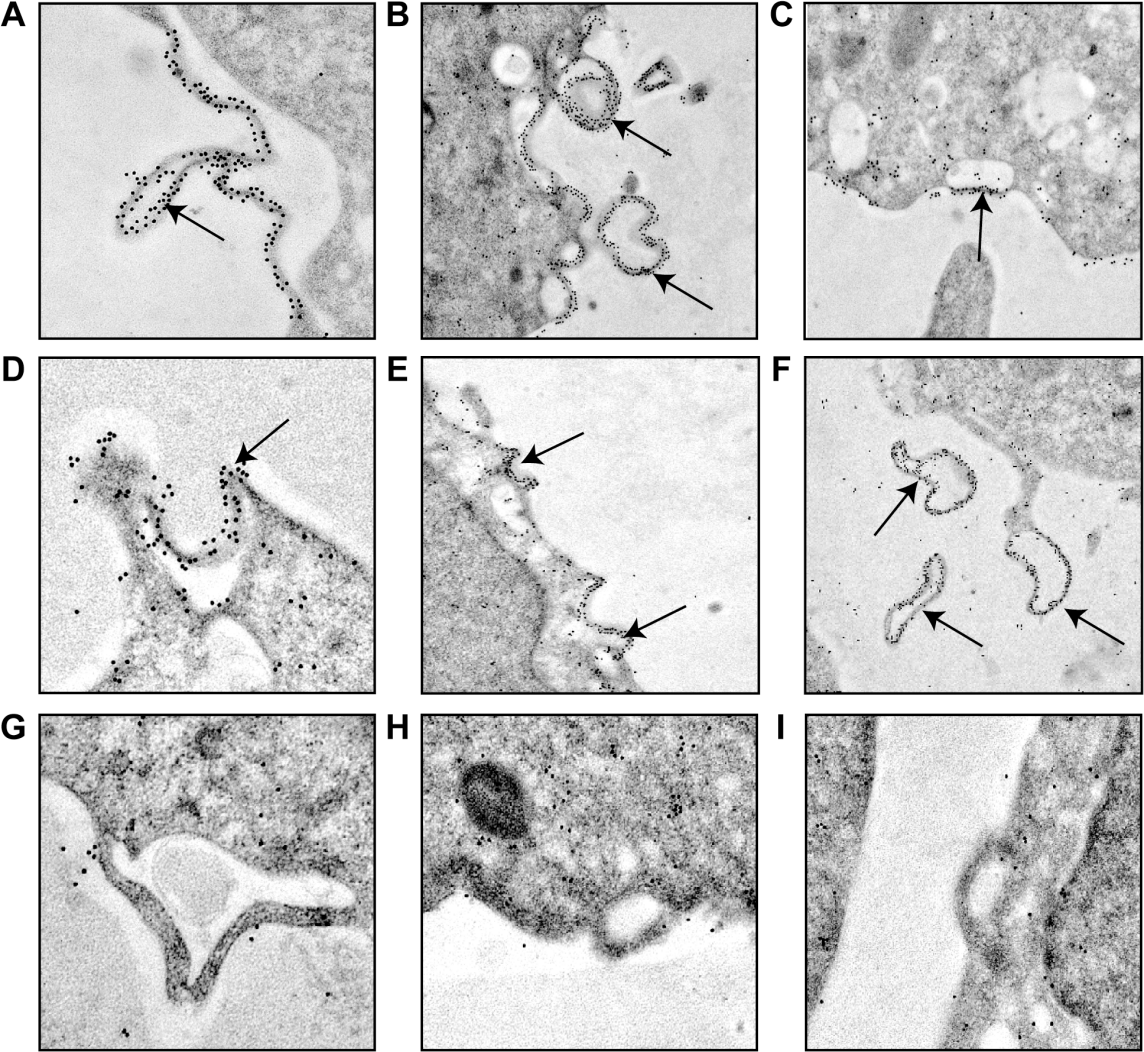
Induction of vesicle budding from the plasma membrane by VSV M proteins. BHK-T7 cells were transfected for 2 h with pTM1 encoding M1 (A-F), pTM1 empty (G), M2 (H) or M3 (I). Cells were fixed 6 h after transfection and immunodetection of VSV M proteins was performed using specific monoclonal antibodies and the corresponding mouse-secondary antibodies coupled to gold particles. Cells were visualized with a transmission electron microscope. Arrows indicate sites of vesicle budding at the plasma membrane (A-E) where M1 protein is concentrated, as well as vesicles already released from the cells (F). Statistical analyses of the gold granules distribution was carried out by unpaired (two-tailed) Student *t*-test. p < 0.01 using the Stata Program Version 11.0.

**Fig 5:**
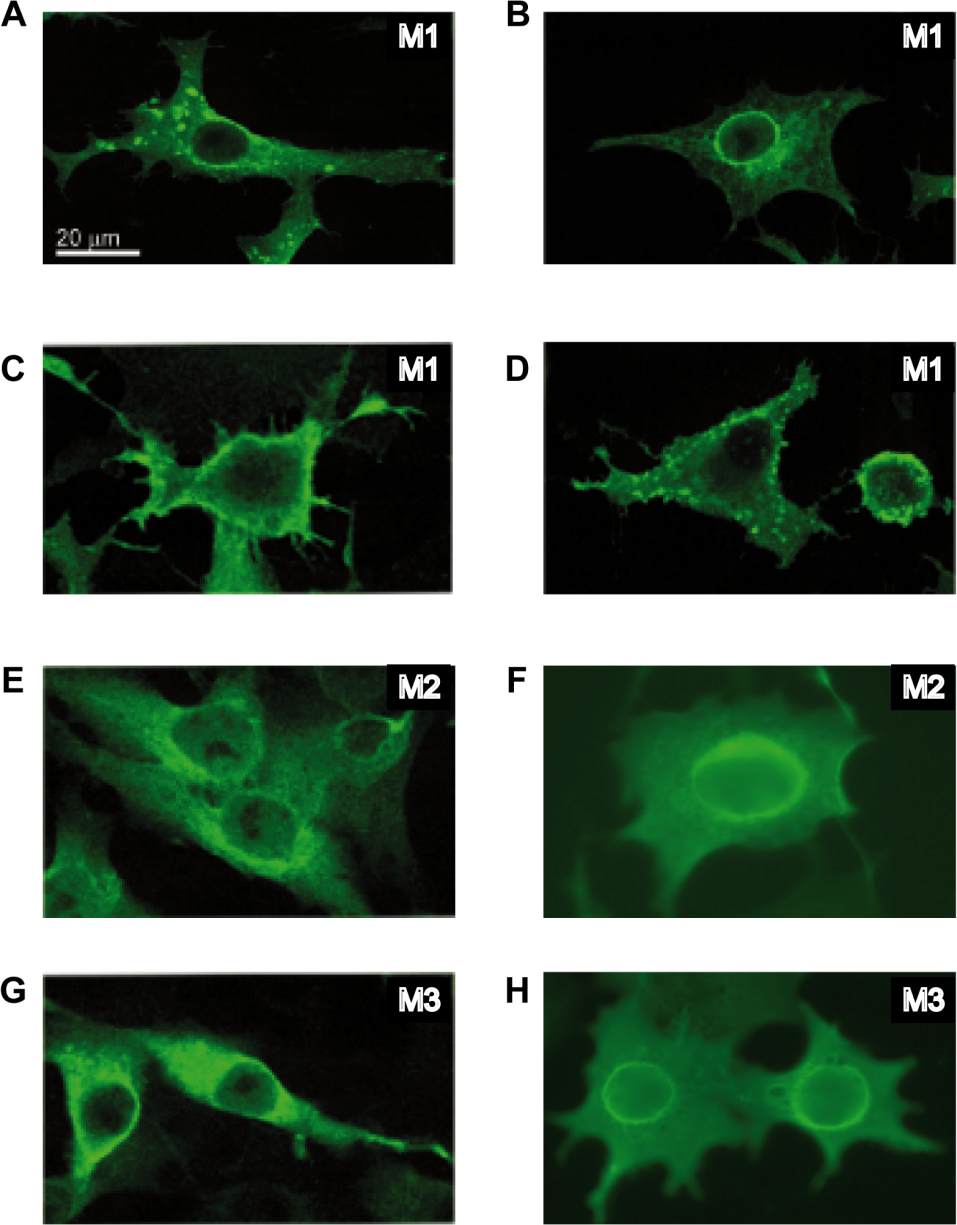
Subcellular localization of VSV M proteins. BHK-T7 cells were transfected for 2 h with pTM1 encoding VSV M1 (A-D), M2 (E-F) or M3 (G-H), and fixed 6 h later. Expression and localization of viral proteins were analyzed by immunofluorescence using specific monoclonal antibodies against M1 that also recognize M2 and M3, and the corresponding mouse secondary antibody conjugated to Alexa 488. Localization of M1 in intracellular membranes and dot-like structures at the nuclear envelope or at the cell surface is shown in panels A, B and C, respectively. Localization of M2 and M3 in intracellular compartments (E and G) or surrounding the nucleus (F and H) is shown. Images were acquired with an Axiovert microscope connected to a digital camera.

VSV M1 protein mediates the budding of viral particles at the plasma membrane in VSV-infected cells [31]. Indeed, the PPPY motif located in M1 protein (amino acids 24 to 27) is a functional late-budding domain. However, the downstream PSAP motif (amino acids 37 to 40) does not display such an activity in VSV-infected BHK-21 cells [5, 32–34]. The fact that M2 and M3 differ from M1 in the amino-terminal domain prompted us to compare the ability of the three proteins to induce vesicle formation and budding from the plasma membrane. Thus, BHK-T7 cells were transfected with plasmids encoding M1, M2 or M3 sequences, or an empty plasmid that served as a negative control, and the intracellular location of the proteins was analyzed by immuno-gold labeling and transmission electron microscopy. Unquestionably, M1 was located at the plasma membrane, and was particularly concentrated at sites of vesicle budding where intracellular vesicles were in close proximity (Fig 4A–4F). Quantitation of gold granules per cm of plasma membrane provided the following average values: 1.7, 20, 1.5 and 1.7 for vector alone, M1, M2 and M3, respectively, obtained as the mean values of the measurement of three cells. The gold granules distribution on plasma membrane was statistically significant (p < 0.01) from that cells transfected with empty vector, M2 or M3 compared to cells transfected with M1. Moreover, the visualization of different stages of vesicle formation and egress allowed us to strengthen the concept of the double membrane-nature of the vesicles released from cells expressing M1 protein (Fig 4A–4F) [35]. The self-interaction between the VSV M1 proteins residing at the plasma membrane and, likely, M1 located at the membrane of intracellular vesicles, might function as a “zipper-like” structure, allowing close contact of the two membranes (Fig 4A–4C). Supporting this possibility, we observed a regular and paired distribution of gold particles at regions of the plasma membrane where intracellular vesicles are recruited and subsequent budding occurs (Fig 4B). Additionally, the interaction of M1 with the ESCRT machinery could eventually trigger the scission and final release of the double-membrane vesicles. This suggested that VSV M1 protein exhibits the ability *per se* to induce the budding of vesicles at the plasma membrane (Fig 4A–4F). However, this intrinsic budding activity was not observed when M2, lacking the PPPY motif, or M3, lacking both L-domains, were transfected (Fig 4H and 4I). Although we could observe intracellular vesicles near the plasma membrane, neither M2 nor M3 were detected at those sites or at the cell surface. Since these findings indicated that VSV M proteins have a diverse localization, we used immunofluorescence to determine their subcellular localization. Results showed that VSV M1 protein was detected mainly at the plasma membrane, in intracellular membranes and in dot-like structures, and also at the nuclear periphery (Fig 5). In contrast, M2 and M3 remained in the cytoplasm (Fig 5). Collectively these results indicated that both the localization of VSV M1 protein at the plasma membrane and its intrinsic function to promote budding required the presence of the first 32 residues at its N-terminus, including the PPPY motif. It is therefore unlikely that M2 and M3 are involved in virus budding at the cell surface. Nevertheless, the intracellular localization of these proteins might support their participation in alternative functions described for M1 other than budding.

### Effect of VSV M proteins on translation

**Fig 6:**
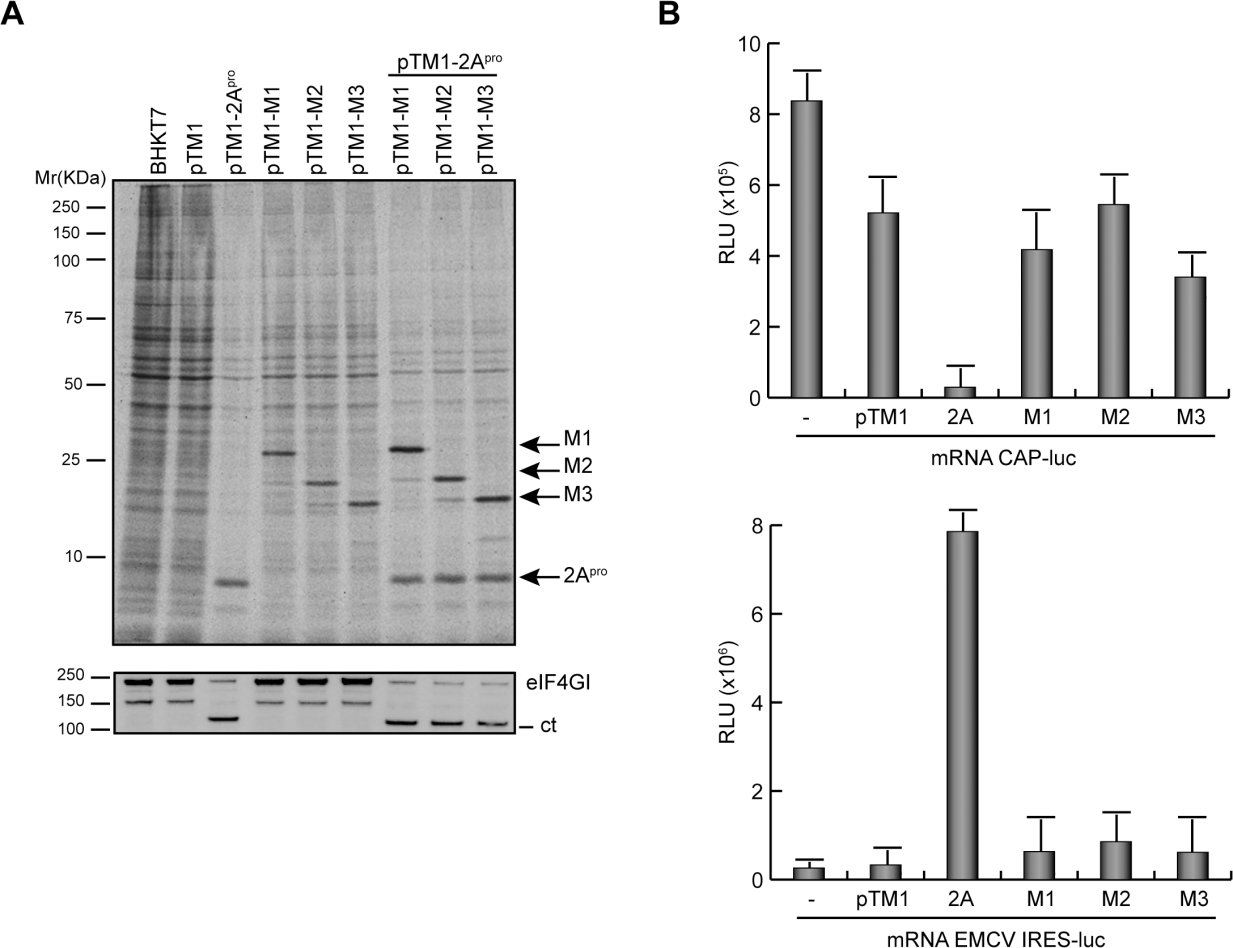
Effect of VSV M proteins on cap- and IRES-dependent translation. (A) Effect of VSV M proteins on cellular translation. BHK-T7 cells were transfected with single pTM1 empty, or pTM1 encoding 2A^pro^ (poliovirus 2A protease), M1, M2 or M3. Additionally cells were co-transfected with pTM1-2A^pro^ as indicated. At 5 hpt, cells were metabolically labelled with [^35^S]Met/Cys for 1 h. Cell lysates were analyzed by SDS-PAGE (17.5%), fluorography and autoradiography (above). Integrity of eIF4GI in the same samples was determined by Western blot using specific antibodies (below). Bands corresponding to the intact eIF4GI and the C-terminal (ct) product resulting from eIF4GI proteolytic cleavage are indicated on the right. Apparent molecular weights are indicated on the left. (B) Effect of VSV M proteins on cap-dependent and cap-independent translation. BHK-T7 cells were transfected for 2 h with pTM1 empty or pTM1 encoding 2A^pro^, M1, M2 or M3 proteins, or left untransfected (-). Transfection medium was removed and cells were incubated with fresh DMEM for 1 h. Then, cells were re-transfected with cap-luc mRNA (mRNA CAP-luc, upper graph) or EMCV(IRES)-luc mRNA (mRNA EMCV-luc, lower graph) for 2 h. Finally, cells were harvested, lysed and luciferase activity was measured as described. Mean values from at least three independent experiments are represented. Error bars indicate standard deviation. (RLU, relative light units).

Because VSV M1 has been shown to block host gene expression, we wished to assess the potential involvement of M2 and M3 proteins in this process. Initially, the impact of the three proteins on mRNA translation was measured. Since our previous findings showed that cytotoxicity induced by VSV M proteins increased in a time-dependent manner, we reduced the time of expression of VSV M proteins in order to analyze their effect on endogenous cellular translation. As a positive control for this assay PV 2A^pro^ was used as representative viral protein that inhibits cap-dependent translation of mRNAs by cleavage of eIF4GI with a concomitant stimulation of translation driven by picornavirus IRESs [36]. To study the impact of VSV M proteins on translation, BHK-T7 cells were transfected with pTM1 plasmids and cells were metabolically labeled at 5 hpt to measure protein synthesis. As anticipated, a clear shut-off of cell translation accompanied by the efficient cleavage of eIF4GI was observed in cells expressing PV 2A^pro^ (Fig 6A). The stimulatory effect of PV 2A^pro^ on translation of uncapped mRNAs was also evident upon co-transfection of pTM1 plasmids encoding the different M proteins and containing the EMCV-IRES. In this case, the synthesis of M proteins was more efficient than in the absence of PV 2A^pro^ (Fig 6A). The expression of VSV M proteins only partially inhibited the synthesis of cellular proteins, with a 30% reduction relative to cells transfected with empty plasmid (Fig 6A). However, this effect was not comparable to the inhibition observed with PV 2A^pro^ (Fig 6A). These results indicated that not only VSV M1 but also its shorter forms, M2 and M3, exhibit inhibitory activity on host cell gene expression. Although a direct inhibition of protein synthesis by M1 has been proposed, it is still unclear whether this is due to the blockade of mRNA transcription and/or export to the cytoplasm. This uncertainty prompted us to investigate whether VSV M1, M2 and M3 proteins directly inhibit the translation of exogenous mRNAs. Thus, BHK-T7 cells expressing different M proteins or PV 2A^pro^ were transfected with reporter mRNAs encoding luciferase and containing either a cap structure or the EMCV-IRES at the 5’ end. The impact of the viral proteins on cap-dependent or IRES-driven translation was then analyzed by determining luciferase activity. As shown in Fig 6B, PV 2A^pro^ inhibited cap-dependent translation of luciferase whereas it stimulated IRES-driven luciferase synthesis. In contrast, VSV M proteins did not significantly impair either cap-mediated or IRES-dependent translation of luciferase compared with cells transfected with empty plasmid (Fig 6B). These findings argue against a direct mechanism of inhibition of cell translation by VSV M proteins.

### Trafficking of mRNAs from nucleus to cytoplasm in BHK cells expressing VSV M proteins

**Fig 7:**
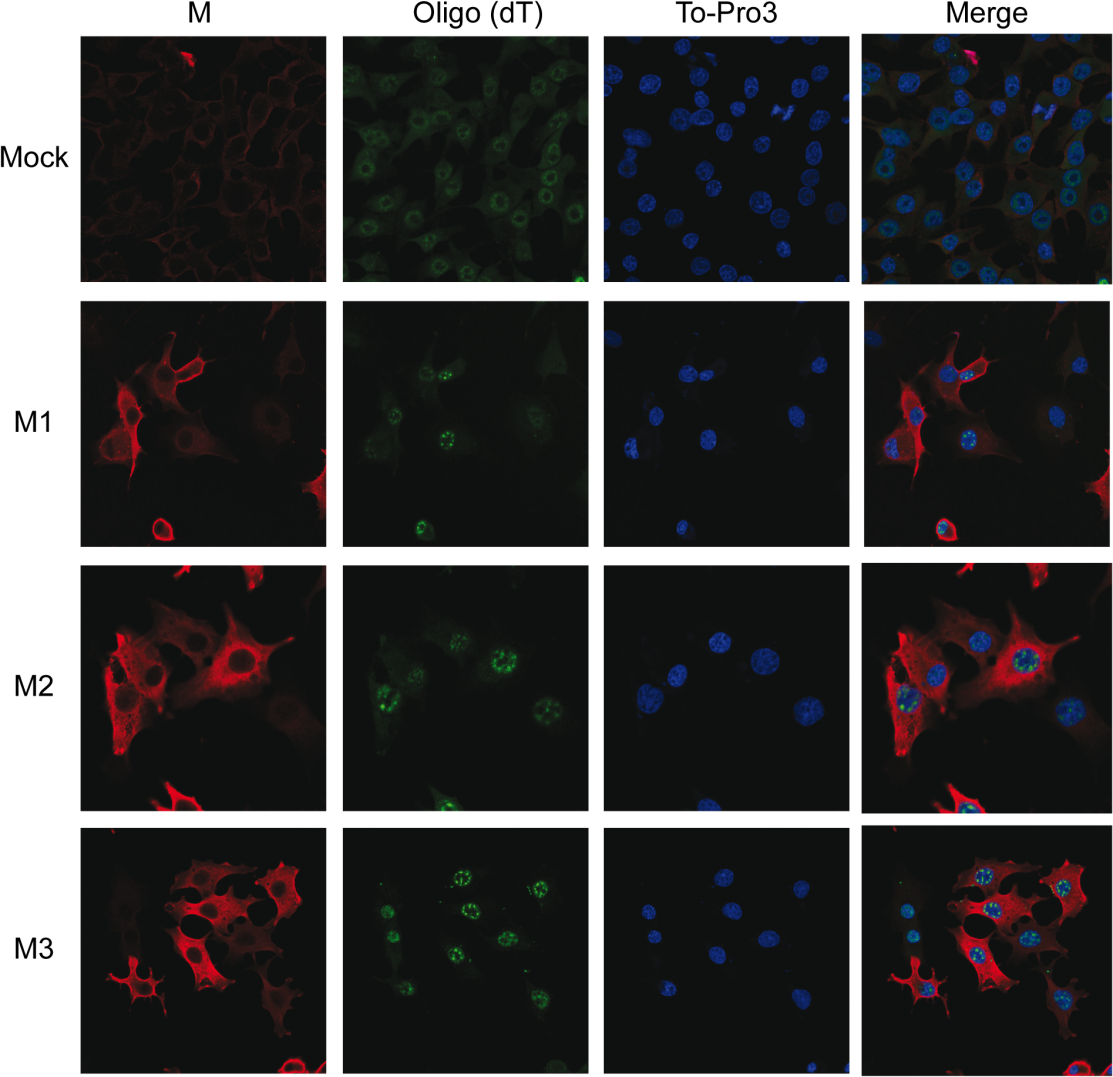
Effect of VSV M2 and M3 expression on nucleus-cytoplasm transport of mRNAs. BHK-T7 cells were transfected for 2 h with pTM1 empty (mock) or pTM1 encoding M1, M2 or M3 proteins. Cells were fixed 6 hpt and *in situ* hybridization with fluorescein-labeled oligo (dT) probe was carried out to detect cellular mRNAs. VSV M proteins were visualized by immunofluorescence using specific monoclonal antibodies against M1 (αM) and the corresponding mouse secondary antibody conjugated to Alexa 555. To-Pro3 was used as a nuclear marker. Images were acquired with a confocal microscope. Merged images are shown on the right.

Given the above results, we questioned whether VSV M proteins could interfere with processes preceding mRNA translation, such as export of RNAs from the nucleus to the cytoplasm. It is recognized that VSV M1 protein inhibits nuclear-cytoplasmic transport of cellular mRNAs and U snRNA when expressed in cells in the absence of other VSV proteins [12, 13, 16, 17]. To address the possibility that M2 and M3 proteins could have similar effects on cellular transport, an *in situ* hybridization assay was employed using a fluorescein-labeled oligo (dT) probe that hybridizes with the poly (A) tail of cellular mRNAs. BHK-T7 cells were transfected with plasmids as before and hybridization of oligo (dT) was carried out after cell fixation at 6 hpt. As expected, the nuclear fluorescein-labeled oligo (dT) signal was increased in the presence of M1 compared with pTM1 control-transfected cells, indicating that the nuclear accumulation of mRNAs in BHK cells was due to the blockade of their export to the cytoplasm (Fig 7, upper panels). Interestingly, the oligo (dT) signal was also concentrated in the nucleus of cells expressing M2 or M3 protein (Fig 7, lower panels). These results suggest that all three VSV M proteins exhibit similar inhibitory effects on the trafficking of cellular mRNAs and reveals that the intact N-terminal domain of VSV M1 is dispensable for blocking nuclear-cytoplasm mRNA trafficking.

### Redistribution of the splicing factor hnRNP H in cells expressing VSV M proteins

**Fig 8:**
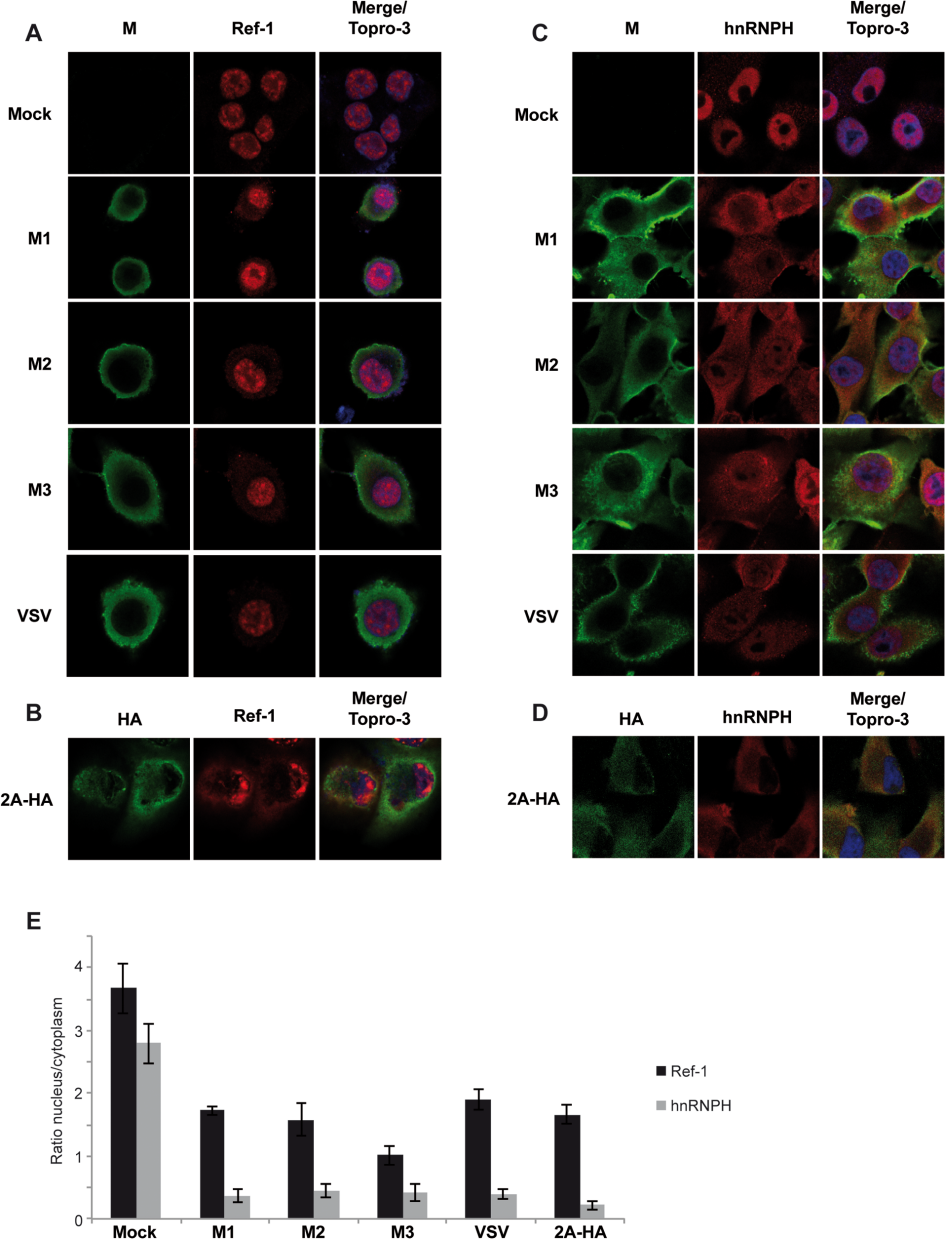
Redistribution of splicing factors Ref-1 and hnRNP H in cells expressing VSV M proteins. BHK-T7 cells were infected with VSV or transfected for 2 h with pTM1 empty (mock) or pTM1 encoding a tagged version of 2A^pro^ (2A-HA), M1, M2 or M3. Cells were fixed at 6 hpi or 6 hpt and immunofluorescence analysis was performed using specific antibodies against VSV M and Ref-1 (A), HA-tag and Ref-1 (B), VSV M and hnRNP H (C) or HA-tag and (D). To-Pro3 was used as nuclear marker. Images were acquired with a confocal microscope. Merged images are shown on the right. E) Quantitation of the nucleus/cytoplasm distribution of Ref-1 (black bars) and hnRNP H (grey bars). Ref-1 and hnRNP H fluorescence intensity was measured in M1-, M2-, M3-, 2A-HA-positive or VSV infected cells using the Image J software. Nucleus/cytoplasm ratios are indicated as the mean values of the measurement of three cells. Error bars indicate standard deviation. Significance of the difference between ratios of mock cells and the indicated samples was assessed by unpaired (two-tailed) Student *t*-test. p < 0.01 (Stata Program Version 11.0).

Aside from the negative effect of VSV M1 on host RNA transcription and mRNA export, it has been reported that VSV infection induces a cytoplasmic relocation of cellular heterogeneous nuclear ribonucleoproteins (hnRNPs), which are host factors involved in different nuclear functions including alternative splicing of immature mRNAs [12, 16, 17, 37]. Nevertheless, this activity has not been assigned to any VSV protein. Relocation of splicing factors might represent an additional viral strategy to indirectly block cellular translation. Thus, we examined whether M1 or its shorter forms, M2 and M3, had the capacity to redistribute nuclear proteins during viral infection. Since PV 2A^pro^ was recently described to disrupt the distribution of splicing factors between the nucleus and the cytoplasm [38, 39], it was included as a positive control. First, we analyzed the effect of VSV M proteins and VSV infection on the distribution of RNA and export factor binding protein (Ref-1 or Aly), an hnRNP that participates in mRNA transcription and transport [40]. In mock transfected cells, Ref-1 exhibited a nuclear distribution pattern that overlapped with the fluorescent nuclear dye To-Pro3 (Fig 8A) [38]. In contrast, in 2A-HA expressing cells, Ref-1 partly relocalized to the cytoplasm (Fig 8B). A similar localization pattern of Ref-1 was observed upon individual expression of M proteins or in VSV infected cells (Fig 8A). To corroborate this result, we analyzed the distribution of a second nuclear splicing factor, hnRNP H, under identical experimental conditions (Fig 8C). As with the previous finding, hnRNP H was localized to the nucleus of mock infected cells (Fig 8C, upper panels), and was partly relocated to the cytoplasm after expression of M proteins, and also in VSV infected cells (Fig 8C). As expected, a dramatic relocation of hnRNP H was achieved by expression of PV 2A^pro^ (Fig 8D). Quantitation of the distribution of Ref-1 and hnRNP H between nucleus and cytoplasm showed that the nuclear/cytoplasm distribution ratios obtained from cells either expressing M proteins, PV 2A^pro^ or infected with VSV were reduced and statistically different (p < 0.01) from that of control cells transfected with empty vector (Fig 8E). These results confirm the participation of M protein in the relocalization of different hnRNPs as proposed previously in studies using a VSV M1 variant [37]. Taken together, these observations provide strong evidence that the individual expression of each VSV M protein is able to modify the nuclear localization of hnRNPs during VSV infection, and establish a new function of VSV M2 and M3 proteins that is shared by M1.

## Discussion

Viruses have developed different strategies to maximize the coding capacity of their genomes. The use of non-canonical mechanisms of translation initiation represents a good example of how multiple viral proteins can be synthesized from a single molecule of viral mRNA. These mechanisms are also exploited by distinct viruses to abrogate host cell translation [41], and have been previously described for different mRNAs from animal viruses, including the M mRNA of VSV, in addition to certain cellular mRNAs [42]. The VSV M mRNA is translated as a polyprotein (i.e. M1, M2 and M3) although only the function of the full-length M1 protein has been studied in depth. In this work, we compared the function of VSV M2 and M3 proteins to that ascribed for M1.

The translation of VSV M mRNA resulted in an unequal and higher proportion of M1 versus M2 and M3 independently of the expression system employed. This phenomenon was more notable in VSV infected BHK cells, where the abundance of the smaller M proteins correlated inversely with the distance between the first AUG start codon and the corresponding internal initiation codons. Considering the leaky scanning mechanism proposed by Jayakar et al., the internal initiation of M2 and M3 translation was presumably achieved by a reduced pool of ribosomes that bypass the first and the second AUG codons [8], by a mechanism that should be independent of the eIF4F complex [43].

This difference in the expression levels of VSV M proteins, although relevant in a physiological context during VSV infection, makes a comparative study of their function difficult. For this reason, we examined the role of M2 and M3 proteins individually synthesized from independent mRNAs. Interestingly, translation of M3 was also observed from M2 mRNA, although in a reduced quantity compared with M2 translation or when it was synthesized from M3 mRNA. Similar to M1, expression of M2 and M3 proteins in BHK cells induced cytotoxicity, which was evident by time-dependent induction of cell rounding, and eventual cell death. These data support the participation of M2 and M3 in VSV-induced cytopathogenesis, and validate the suitability of our expression system for the study of VSV M protein function [8].

Analogous to other multifunctional proteins encoded by animal viruses (e.g. viral proteases), VSV M1 protein plays essential roles during the virus replicative cycle and at the same time interferes with host cell pathways that are deleterious for the establishment of a productive infection. In addition to its involvement in virus assembly and release, VSV M1 also promotes budding of intracellular vesicles at the plasma membrane in the absence of other viral proteins [44–46]. We found that in contrast to viroporins, a family of pore-forming viral membrane proteins that contribute to virus budding, the mechanism of budding mediated by VSV M1 did not involve an increase in plasma membrane permeability [44–46]. Moreover, M2 and M3 proteins did not exhibit viroporin-like activity or plasma membrane localization, and did not induce vesicle budding. These findings confirm that the first 30 amino acids, which are absent in M2 and M3, selectively confers M1 with an ability to promote budding, and are required for its transport to the plasma membrane [44–46]. Therefore, it is unlikely that M2 and M3 participate in the budding of VSV particles. However, all three M proteins showed a perinuclear distribution and equally affected host cell translation. We explored the possible mechanisms that account for this inhibition and found that VSV M proteins do not directly block cellular translation. Several observations are consistent with this assumption. The integrity of the translation initiation factor eIF4GI, a target of viral proteases from certain RNA viruses (e.g. 2A from picornaviruses, HIV protease) which induce direct shut-off of cell protein expression [47, 48], was not altered by VSV M proteins. Moreover, translation inhibition mediated by M proteins was not equivalent to the profound block exerted by PV 2A^pro^. Importantly, in contrast to endogenous mRNAs, the translation of exogenous mRNAs containing either a cap structure or an IRES was not impaired in cells expressing the VSV M proteins, whereas PV 2A^pro^ suppressed translation in the former case and stimulated cap-independent translation in the latter. These results indicate that not only VSV M1 but also M2 and M3 proteins interfere with the preceding steps of gene expression rather than on the translation machinery. It is well established that VSV M1 inhibits host transcription [9, 10] as well as nuclear-cytoplasmic RNA transport, which is dependent on its interaction with the RaeI/Nup98 complex [12, 13, 16, 17]. Recently a mechanism of inhibition has been proposed from crystallography studies of the M/RaeI/Nup98 complex [49]. M1 mimics the phosphate backbone of nucleic acids and therefore competes with host cell mRNAs for binding to RaeI. Importantly, biochemical analysis revealed that the finger region of M1 protein (residues 49–61), and a highly conserved Met51 within it, blocked RaeI RNA binding activity [49]. Our present findings showed that VSV M2 and M3 proteins also block host cell mRNA export. Similar to the mode of action of M1, the finger domain present in M2 might provide the binding site to RaeI and Nup98. Moreover, the fact that M3 (residues 51–229) lacks the first amino acids of the finger but still displayed inhibitory function on mRNA export, suggests that M3 meets the minimal and essential structural features to mimic M1 function. However, further studies are needed to confirm the effect of VSV M2 and M3 on RaeI/Nup98 function.

An additional level of repression of host gene expression mediated by M1 during VSV infection is the impairment of mRNA transcription or/and its export by the relocalization of hnRNPs to the cytoplasm. We found that the splicing factors Ref-1 and, more remarkably, hnRNP H, were redistributed to the cytoplasm in VSV-infected cells, and this phenomenon was reproduced by VSV M proteins expressed alone. Previous studies reported that hnRNP A1 export induced by VSV is also dependent on Rae1 and correlates with a delayed induction of apoptosis by VSV, but not with host cell shut-off [37]. The recent finding that the VSV M1 mutant M51R, defective at blocking host transcription, failed to promote hnRNP A1 relocalization, strongly supports the participation of VSV M2 and M3 proteins in the inhibition of hnRNP export [37]. This observation also underscores the importance of Met51 and thus the potential implication of RaeI in the redistribution of nuclear factors induced by VSV M2 and M3.

In summary, we describe novel functions of two additional matrix proteins, M2 and M3, expressed during VSV infection by alternative initiation of M mRNA. These viral proteins, although deprived of the M1 budding activity, might contribute, to a different degree, to M1-associated cytopathogenicity during VSV infection. We also provide the first evidence that VSV M2 and M3 suppress cell gene expression by interfering with nucleus-cytoplasmic mRNA export and the localization of some splicing factors, leading to an eventual defect in cell host protein synthesis. Finally, we hypothesize that in a physiological context, M2 and M3 could relieve M1 of its common function to facilitate M1-mediated assembly and budding of new viral particles. This redundancy of functions exhibited by VSV matrix proteins may represent an advantageous strategy not only for the establishment of VSV replication but also to ensure multiple cycles of infection in the host cell.

## Materials and Methods

### Cell culture and virus stock

Baby hamster kidney (BHK-21 and clone BSR-T7/5, designated as BHK-T7) cells [50] were used in this work. Cells were grown at 37°C in Dulbecco’s Modified Eagle’s Medium (DMEM) supplemented with 5% or 10% foetal calf serum (FCS) and non-essential amino acids. BHK-T7 cells were additionally cultured in the presence of 2 mg/ml geneticin (G418, Sigma) on every third passage. Cells were infected with VSV (Indiana strain) at a multiplicity of infection of 10 pfu (plaque forming units) /cell.

### Plasmids

The pTM1-derived expression plasmids containing the coding sequences of VSV M1, M2 or M3 were constructed using the pBS-GMMG plasmid as a DNA template (kindly provided by Dr. Whitt, University of Tennessee, USA) with the forward primers 5´NcoIM1 (GCGCGCCCATGGGCAGTTCCTTAAAGAAGATTCTCG), 5´NcoIM2 (GCGCGCCCATGGAGTATGCTCCGAGCGCTCCAATTG), 5´NcoIM3 (GCGCGCCCATGGACACCTATGATCCGAATCAATTAA), respectively, and the reverse primer 3´BamHI (GCGCGCGGATCCTTACTAGCTCATTTGAAGTGGCTGATA). PCR products were digested with NcoI/BamHI restriction enzymes and inserted into the corresponding sites of pTM1. The pTM1-2A^pro^, pTM1-2B and pTM1-2C plasmids have been previously described [51–53]. The construct pKS-luc and pTM1-luc have been described [54]. A SV replicon, pT7repC+M1, was obtained by cloning the NdeI/BamHI-digested PCR fragment encoding the M1 protein after the sequence of SV capsid protein (C), into the corresponding sites of plasmid pH3′ 2J-C [55]. In a second step, the fragment digested with AatII and XhoI was inserted into the pT7SVwt vector [55]. Replicons repC and repC+6K have been described [55, 56]. Nucleotide sequences of all constructs were verified using standard sequencing procedures.

### Transfection of mammalian cells

BHK-T7 cells were transfected with 1μg pTM1-based expression vectors using Lipofectamine 2000 (Invitrogen). Alternatively, cells were co-transfected with a combination of two plasmids (2 μg in total). For RNA transfection, 2 μg of mRNA plus 2 μl Lipofectamine 2000 in Opti-mem I medium (Invitrogen) were added per well and incubated for 2 h at 37°C. Then, the transfection mix was removed, and the cells were supplemented with fresh medium containing 5% FCS. BHK-21 cells were transfected by electroporation with SV-derived replicons or mRNAs obtained by *in vitro* transcription, using the corresponding linearized DNA plasmids as templates. The pKS-luc plasmid was used as a template to obtain cap-luc mRNA. Transcription reactions including the cap analog, m^7^G (5´)ppp(5´)G, were carried out with T7 RNA polymerase (Promega). *In vitro* transcription reactions using linearized pTM1-derived plasmids did not require the addition of cap analog. Subconfluent BHK cells were electroporated as described previously [29].

### *In vitro* translation

*In vitro* translation was carried out in rabbit reticulocyte lysates (RRL, Promega) in the presence of EasyTag^TM^ EXPRESS ^35^S Protein Labeling mix, [^35^S]Met/Cys (Perkin Elmer). In brief, 100 ng of the corresponding mRNA obtained by *in vitro* transcription was added to the reaction mixture and incubated for 90 min at 30°C. To analyze protein synthesis, samples were diluted in sample buffer, boiled for 5 min and subjected to SDS-PAGE (15%), followed by fluorography and autoradiography.

### Analysis of protein synthesis in BHK cells

Protein synthesis was analyzed at the indicated times by replacing the growth medium with DMEM without methionine/cysteine and supplemented with [^35^S]Met/Cys. Cells were collected 45 min later in sample buffer and analyzed as described before. Protein synthesis was quantified by densitometry of the bands of interest using a GS-800 Calibrated Densitometer (Bio-Rad).

### Western blotting

After SDS-PAGE, proteins were transferred to a nitrocellulose membrane as described [57]. To detect VSV M proteins, a specific mouse monoclonal antibody, 23H12 clone (generously provided by Dr. Lyles, Wake Forest School of Medicine, North Carolina, USA) was used at 1:6000 dilution. Incubation with primary antibodies was performed for 2 h at 4°C, and the membrane was washed three times with PBS containing 0.2% Tween-20 and incubated for 1 h with horseradish peroxidase-conjugated anti-mouse antibodies (Promega) at 1:5000 dilution. Finally, the membrane was washed three times and bound antibodies were detected using the ECL detection system (Amersham).

### Measurement of luciferase activity

Cells transfected with the indicated mRNAs containing the luciferase reporter were lysed in a buffer containing 25 mM glycylglycine (pH 7.8), 0.5% Triton X-100 and 1 mM dithiothreitol. Luciferase activity was determined using the *luciferase assay system* (Promega) and a Mononlight 2010 apparatus (Analytical Luminescence Laboratory) as described [47, 58].

### Membrane permeabilization assay

Transfected cells were seeded in wells of an L-24 plate. At the indicated times, cells were pretreated with 0.5 mM hygromycin B (HB, Clontech) for 15 min at 37°C, or left untreated. Then, proteins were labelled for 40 min with 10 μCi [^35^S]Met/Cys (Promix; Amersham Pharmacia) in methionine/cysteine-free DMEM in the presence or absence of 0.5 mM HB. Subsequently, cells were collected in sample buffer, boiled for 4 min and protein synthesis was analyzed as described. Protein synthesis was quantified by densitometry of either the band corresponding to the SV capsid protein (C) or a cellular protein, and was calculated by dividing the values obtained for samples treated with HB by the corresponding values obtained from untreated cells.

### Immunofluorescence microscopy and fluorescence in situ hybridization

Fixation and permeabilization of BHK-transfected cells were performed as described [29]. Cells were examined with a confocal LSM510 lens coupled to an Axio Imager Z1 microscope (Zeiss) with a 63x/1.4 oil Plan-Apochromat objective. Image processing was performed with Huygens 3.0 software (Scientific Volume Imaging). The following primary antibodies were used: VSV M-specific mouse monoclonal antibody 23H12 clone (1:800), rabbit antisera against REF-1/Aly (a generous gift from Elisa Izaurralde, Max Planck Institute for Developmental Biology, Tübingen, Germany) and hnRNP H (Abcam), both diluted 1:100. Specific antibodies conjugated to Alexa 488 or Alexa 555 (Invitrogen) were used as secondary antibodies at 1:500 dilution. To-Pro-3 (Invitrogen) diluted 1:1000 was used to stain nuclei. Detection of polyadenylated mRNAs by fluorescence *in situ* hybridization (FISH) was carried out with fluorescein-labeled oligo (dT) (Gene link). Cells were fixed with 4% paraformaldehyde (PFA) for 15 min at RT, permeabilized with 0.1% Triton in PBS for 10 min and then subjected to three washes: first, 1× phosphate-buffered saline (PBS), second, 1× PBS and 1× saline-sodium citrate buffer (SSC), and third, 2× SSC. Cells were then incubated at 37°C with pre-hybridization buffer (2× SSC, 20% deionized formamide, 0.2% BSA and 1 mg/ml yeast tRNA). Subsequently, cells were incubated at 37°C for 4 h with hybridization buffer (2× SSC, 20% deionized formamide, 0.2% BSA, 1 mg/ml yeast tRNA, 10% dextran sulphate and 1 pmol/μl oligo (dT) probe). Preparations were then washed four times at 42°C for 5 min: the first wash was performed with 2× SSC and 20% formamide; the second with 2× SSC; the third with 1× SSC and 1× PBS; and the last wash with 1× PBS.

### Electron microscopy

Transfected cells were processed for electron microscopy as follows: at 6 hpt, cells were fixed with 2% glutaraldehyde in 0.2 M HEPES buffer, pH 7.4, for 1 h at room temperature and immediately scraped off from the plate. Cells were then washed twice and resuspended in 0.2 M HEPES buffer, pH 7.4. After fixation, cells were dehydrated and infiltrated with resin. Thin sections were obtained and collected on nickel grids. The grids were then incubated for 1 min in TBS buffer (30 mM Tris-HCl [pH 8.2] containing 150 mM NaCl), followed by 5 min incubation in TBG buffer (TBS containing 0.1% BSA and 1% gelatin). For immunodetection of VSV M proteins, M-specific primary antibody was added in TBG buffer and incubated for 1 h. Subsequently, the grids were washed three times with TBS containing 0.1% BSA followed by incubation with mouse-specific secondary antibodies bound to 10 nm-gold particles for 1 h. Finally, grids were washed and examined with a JEM1010 (Jeol) transmission electron microscope equipped with a 4K x 4K TemCam-F416 digital camera (TVIPS GmbH, Gauting, Germany).
